# The Importance of Exercise in Alzheimer’s Disease and the Minds in Motion^®^ Program: An Editorial

**DOI:** 10.3390/jfmk5030059

**Published:** 2020-08-04

**Authors:** Jessica Watson, Nathan O’Keeffe, Sarah L. West

**Affiliations:** 1 Department of Psychology, Trent University, Peterborough, ON K9L 0G2, Canada; jessicawatson@trentu.ca; 2 Department of Biology, Trent University, Peterborough, ON K9L 0G2, Canada; nathanokeeffe@trentu.ca; 3 Trent/Fleming School of Nursing, Trent University, Peterborough, ON K9L 0G2, Canada

**Keywords:** Alzheimer’s Disease, dementia, physical activity, exercise, cognition, quality of life, Minds in Motion^®^, The Alzheimer’s Society of Canada

## Abstract

Alzheimer’s Disease (AD) and other dementias are prevalent neurodegenerative diseases characterized by decreased cognition, physical function, and quality of life. Currently, millions of people are living with AD and other dementias. With no cure, research has examined the use of non-pharmacological treatment options including exercise. Many high-quality studies demonstrate that physical activity slows the progression of AD’s many outcomes, and is beneficial to overall quality of life in those living with AD. However, creating exercise interventions at the community level that individuals will adhere to is often a challenge. The Alzheimer’s Society of Canada developed a unique program that combines physical activity with cognitive stimulation in a social atmosphere: The Minds in Motion^®^ program. Minds in Motion^®^ addresses many of the barriers often linked to poor physical activity participation in chronic diseases (such as inclusion of the care partner), to ensure the best program uptake. The Minds in Motion^®^ program has anecdotally been successful in helping to increase physical function and social skills in those living with dementia. However, it is important to connect community-driven programs with the academic research community, to create an opportunity for high quality evaluation metrics that can be disseminated at multiple levels: to research audiences, clinical audiences, and to those in the community. With ongoing collaborations between research and community programs, there is a greater opportunity to understand the positive impact of a program, which ultimately increases the chance of funding for the program. In this editorial, we highlight that community-integrated research is an important priority for future collaborations.

Alzheimer’s Disease (AD) is the most common form of dementia and is a progressive neurodegenerative disease that is characterized by many neurological components including behavioral changes, cognitive decline, as well as physical impairments [1]. Approximately 35 million people globally are living with dementia, and the rate is expected to continue to rise each year with an estimated 66 million diagnosed with dementia by the year 2030 [1]. In Canada, approximately 340,000 individuals were diagnosed with AD in 2011 [2]. Due to the increased number of individuals living with AD, there is a substantial burden on healthcare services [1]. Perhaps more importantly, AD results in a reduction in an individual’s quality of life including psychological well-being, which can lead to poor health and well-being in this population [3,4]. Thus, managing the symptoms and outcomes associated with all forms of dementia, including AD, is of high importance.

It is well known that AD has many symptoms associated with cognitive decline including poor memory, judgement, orientation, reasoning, as well as behavioral changes such as frustration and depression. Reductions in physical function are also common [1,5]. Characteristic loss of physical function in AD may include experiences of extreme fatigue, reduced gait speed, balance, muscle strength, muscle structure, and dexterity [6,7,8]. These changes can make it difficult to participate in daily activity and exercise. However, an interesting question emerges: does AD lead to poor physical function and/or poor physical activity participation? Or does reduced physical activity participation precede the diagnosis of AD? It is likely a combination of the two scenarios. There is evidence that poor muscle strength (measured by handgrip) and slow gait speed are good predictors of AD development in future years [9,10,11]. This data suggests that poor indices of strength and physical function occur prior to AD diagnosis. Further to this, recent research demonstrates that increased body weight (i.e., obesity) or abdominal obesity are associated with an increased incidence of all types of dementia (AD included) [12]. Thus, it is conceivable that the prevention of obesity via healthy lifestyle choices including physical activity may help to prevent dementia onset. That said, diagnosis of AD is associated with a subsequent decline in physical function [1,13], therefore it may be a circular relationship whereby obesity, poor strength/gait are predictive of dementia onset, and that dementia (including AD) leads to further exacerbation of poor physical function, activity participation, and physical health.

Pharmaceutical medications such as cholinesterase inhibitors (Aricept, Razadyne, or Exelon) and Memantine (Namenda) can be used to aid in the treatment of AD [14]. However, medication has only proven limited effectiveness and may have unwanted side effects, thus a focus on non-pharmacological areas of treatment is warranted [15,16]. The use of exercise as medicine has gained recent attention from researchers and clinicians [15,16] given the positive impact of exercise programs on various outcomes in chronic diseases such as cardiovascular diseases, cancer, obesity, depression, as well as in persons living with dementia [4,17,18,19,20]. There is strong evidence that supports the use of exercise in AD; exercise has been demonstrated to improve cognition, quality of life and physical function in persons living with AD [8,17,21]. Cardiorespiratory fitness (which improves with exercise) can help increase memory and lower hippocampal atrophy levels, suggesting that exercise can help preserve cognitive function [8,19,22]. Exercise is also associated with improved quality of life and decreased depression in individuals living with AD [17,21]. In terms of physical function, aerobic exercise postpones physical decline by increasing dual task performance (such as walking and carrying a conversation), increasing mobility and balance, along with decreasing fall rates in persons living with AD [1,5,17,23,24,25].

While data clearly demonstrates that exercise is beneficial to those living with dementia and AD, figuring out how to deliver exercise programing at a community level is challenging [26]. There are many factors that may prevent an individual from committing to participating in an exercise intervention including socioeconomic status, gender, age, race, the perceived benefits of attending, and possible lack of encouragement or support [26]. These barriers to exercise participation are further exacerbated in those with a chronic disaese [27,28]. For example, one study conducted semi-structured interviews with individuals with chronic conditions, and found that social/cultural identities, personal support, and the percevied benefits of exercise were significant factors infleuncing exercise class attendance rates [27]. Importantly, having at least one personal supporter was associated with higher levels of attendance [27]. Overall, evidence suggests that behavioral barriers such as low self-efficacy [29] are externally valid barriers that impede exercise particpation in those with chronic disease. Therefore, the importance of community based exercise programs that provide support and also address behavioral and emotional barriers will likely be the most successful in maximizing adherence, enjoyment and effectiveness.

In 2009 The Alzheimer’s Society of Canada developed an innovative program named Minds in Motion^®^ (MiM), targeted towards persons living with symptoms of early to mid-stage dementia. So, what exactly is MiM? MiM is an eight week program that combines physical activity and mental stimulation into a two hour session once per week [30]. The instructor of the MiM program also provides ‘homework’, where participants are provided exercises to complete at home. The instructor completes a verbal check-in to reinforce the importance of the at home exercises [30]. The physical activity program is gentle and easy, and focuses on building mobility, balance, and flexibility. It is completed in a social environment with others, so it increases connectivity, and confidence as well [30]. The program is open to all seniors within the local community who are living with dementia and is currently being offered in a variety of locations across Canada, including British Columbia, Alberta, Northwest Territories, Manitoba, Ontario, and Prince Edward Island. MiM was designed to address the needs of seniors with a variety of backgrounds (e.g., variety of cultures, indigenous communities, learning styles, and both mental and physical abilities). Another important aspect of the MiM program is that for those living with dementia, their care partner or additional partner attend the program sessions as well [30]. Therefore, MiM addresses many of the barriers to exercise participation in chronic disease; it offers a social environment that encourages confidence and connectivity, considers cultural differences and learning styles, and involves care partners as an extra layer of support.

The MiM program has a number of important goals: to increase the number of programs available that provide mental health services and support; to increase the awareness and support to individuals, families, and care partners regarding mental health education; to increase community awareness and partnerships to sustain the MiM initiative; and to improve physical funcitoninig and general well-being of program participants. Anecdotally, and thorugh pilot data, the MiM program has been well recevied and is an effective program for those living with mild to moderate dementia. Individuals who particpate in the MiM program report improved mental functioning following a MiM session, gains in their confidence and comfort with their life, as well as improved balance, mobility, strength, endurance, and increased flexibility [30].

However, more hypothesis-driven research that demonstrates the impact of MiM on multiple outcomes (physiological, emotional, and psychosocial) is still needed. High-quality research followed by an advanced knowledge-mobilizaton plan that targets medical professionals in the community (for example, via an infographic resource for physicians), research scientists (for example, via research conference presentations), as well as reports to families within communities are ways to effectively disseminate the results. Incorporating high quality research outcomes within a community delivered program such as MiM will provide key evaluation metrics that demonstrate effectiveness (i.e., an evidence-based strategy) in an increasingly evidence-savvy culture. The ultimate goal is to encourage increased program participation and uptake, as well as solidify funding from public and private sectors.

In conclusion, with the incidence of dementia on the rise, there is a continued need to explore and implement non-pharamcological treatment options including exercise [1,4,17,21,23,24,25,31]. It should be noted that while the current discussion focuses on modifying physical activity to improve outcomes in those living with AD, other lifestyle factors such as diet may also be important. For example, certain diets (such as the Mediterranean) have been found to reduce inflammation, risk of cardiovascular disease, and counteract oxidative stress which can underlie AD [32,33,34,35]. However, few studies have examined the combined effect of diet and exercise in AD [36], and this remains an area for future research. In the meantime, implementing the use of exercise as medicine in persons living with AD is important, and community-based programs that address common barriers to exercise program uptake (such as the Minds in Motion^®^ program) are necessary initiatives that lead to an improved quality of life in those living with AD. However, we believe that it is critical for members of the scientific community to join forces with community program teams, and assist with developing and implementing research-driven outcomes that demonstrate program effectiveness. Reliable program effectiveness data increases the opportunity for funding (both public and private), and ultimately increases program availability to more individuals. For example, our research team is commited to working with our local Alzheimer Society (Peterborough, Kawartha Lakes, Northumberland & Haliburton) to assist in creating appropriate evaluation metrics for the above stated reasons. We look forward to continuing to bridge the connection between academic research driven outcomes and community-based programming; and in the end improving care and health outcomes for persons living with dementia.
